# Identification and characterization of roles for Puf1 and Puf2 proteins in the yeast response to high calcium

**DOI:** 10.1038/s41598-017-02873-z

**Published:** 2017-06-08

**Authors:** Ofir Haramati, Anastasia Brodov, Idan Yelin, Avigail Atir-Lande, Nitzan Samra, Yoav Arava

**Affiliations:** Faculty of Biology, Technion – Israel Institute of Technology, Haifa, 32000 Israel

## Abstract

Members of the yeast family of PUF proteins bind unique subsets of mRNA targets that encode proteins with common functions. They therefore became a paradigm for post-transcriptional gene control. To provide new insights into the roles of the seemingly redundant Puf1 and Puf2 members, we monitored the growth rates of their deletions under many different stress conditions. A differential effect was observed at high CaCl_2_ concentrations, whereby *puf1Δ* growth was affected much more than *puf2Δ*, and inhibition was exacerbated in *puf1Δpuf2Δ* double knockout. Transcriptome analyses upon CaCl_2_ application for short and long terms defined the transcriptional response to CaCl_2_ and revealed distinct expression changes for the deletions. Intriguingly, mRNAs known to be bound by Puf1 or Puf2 were affected mainly in the double knockout. We focused on the cell wall regulator Zeo1 and observed that *puf1Δpuf2Δ* fails to maintain low levels of its mRNA. Complementarily, *puf1Δpuf2Δ* growth defect in CaCl_2_ was repaired upon further deletion of the Zeo1 gene. Thus, these proteins probably regulate the cell-wall integrity pathway by regulating Zeo1 post-transcriptionally. This work sheds new light on the roles of Puf proteins during the cellular response to environmental stress.

## Introduction

Post-transcriptional gene regulation relies on the coordinated function of mRNA-binding proteins (mRBPs). This family of proteins is implicated in every aspect of mRNA maturation, transport in the cell, translation and degradation^1^. Recent proteomic studies in yeast, worms and mammalian cells identified many novel mRBPs, which encompass up to 15% of an organism’s proteome^2–6^. Intriguingly, many of these novel mRBPs had been identified previously to have other cellular functions; most notable are enzymatic activities in various metabolic pathways^7, 8^. This suggests that mRBPs also serve as immediate coordinators between the cellular metabolic state and the biogenesis of an mRNA^9^.

The PUF family of mRBPs was first identified in *D*. *melanogaster* (Pumillio protein) and *C*. *elegans* (FBF), and is highly conserved among the eukaryotic species^10, 11^. PUF proteins are characterized by a conserved RNA-binding domain (Pum domain) that consists of a repeating unit (usually eight repeats) of a three α helix motif^12^. PUF proteins can bind hundreds of mRNAs, usually through elements in the 3′UTRs of their targets^13–16^. *S*. *cerevisiae* cells contain six PUF proteins, designated Puf1 to Puf6. Each protein associates with many mRNAs, suggesting that more than 10% of yeast mRNAs are regulated by the PUF family^13^. Importantly, the mRNAs bound by some members are distinct. Puf1 and Puf2 interact preferentially with mRNAs encoding proteins of the cell periphery, in particular membrane-associated proteins^13, 15^. Interestingly, the list of targets of Puf1 is smaller than Puf2, and almost all targets of Puf1 are also bound by Puf2^13^. The two proteins also recognize a similar RNA sequence (dual UAAU motif), which diverges from the canonical PUF recognition motif^17^. Puf3 binds cytoplasmic mRNAs that encode mitochondrial proteins and nuclear proteins^13, 18^, and Puf4 and Puf5 interact selectively with mRNAs encoding nuclear components^13^. A recent high-resolution study further expanded the list of Puf5 targets and identified mRNAs encoding components of the translation machinery and mitochondria^19^.

By binding sets of transcripts with common functions, PUF proteins were suggested to coordinately control mRNAs that are required for a biological process. Molecular studies had characterized a regulatory role for some of these proteins: Puf1, Puf3, Puf4 and Puf5 enhance mRNA degradation^20–23^. Puf2 and Puf3 are involved in the localization of mRNAs^24, 25^. Puf6 represses translation of the *ASH1* mRNA while in transit to its destination^26–28^. Yet, the impact of these functions on cellular physiology is largely unknown. Cells deleted of either one or all Puf proteins show similar growth in rich media, thus suggesting a role only upon unique growth conditions^29^. Indeed, the function of many mRNA targets of Puf proteins is related to stress conditions^30, 31^, and Puf3 was recently shown to regulate expression during glucose depletion^32^. However, none of the Puf deletion strains have yet been shown to be essential for growth under any growth condition.

Herein we aimed at identifying the physiological importance of Puf1 and Puf2. These proteins are particularly puzzling as they share a similar structure that is comprised of only six PUF repeats and an additional RRM motif. This structure is distinct from the other Puf proteins. As indicated above, the two proteins appeared to bind an overlapping list of target mRNAs and were shown to associate with a similar mRNA motif. To resolve this seemingly redundant role, we monitored the growth rates of *puf1Δ* and *puf2Δ* under many different stress conditions. Of significance was the effect of high CaCl_2_ concentrations, which affected the growth of *puf1Δ* to a much larger extent than *puf2Δ*. This effect was exacerbated in the double knockout *puf1Δpuf2Δ*. RNA-seq analyses for strains grown at high CaCl_2_ concentrations identified unique subsets of mRNAs that are likely to be regulated by Puf1 and Puf2. These subsets differed significantly between the proteins. Intriguingly, the most affected genes were not shown previously to associate with Puf1 or Puf2, and were not found to contain a dual UAAU binding sequence. We next focused on a candidate target (*ZEO1*) and observed increased mRNA levels in Puf deletion strains. Further deletion of the *ZEO1* gene from the *puf1Δpuf2Δ* strain restored its growth in high CaCl_2_ media. This suggests a regulatory loop in which Puf1p and Puf2p maintain low levels of Zeo1p, and this low level is important for proper growth upon CaCl_2_ application.

## Results

### Puf deletions differ in sensitivity to high CaCl_2_ concentrations

**Figure 1:**
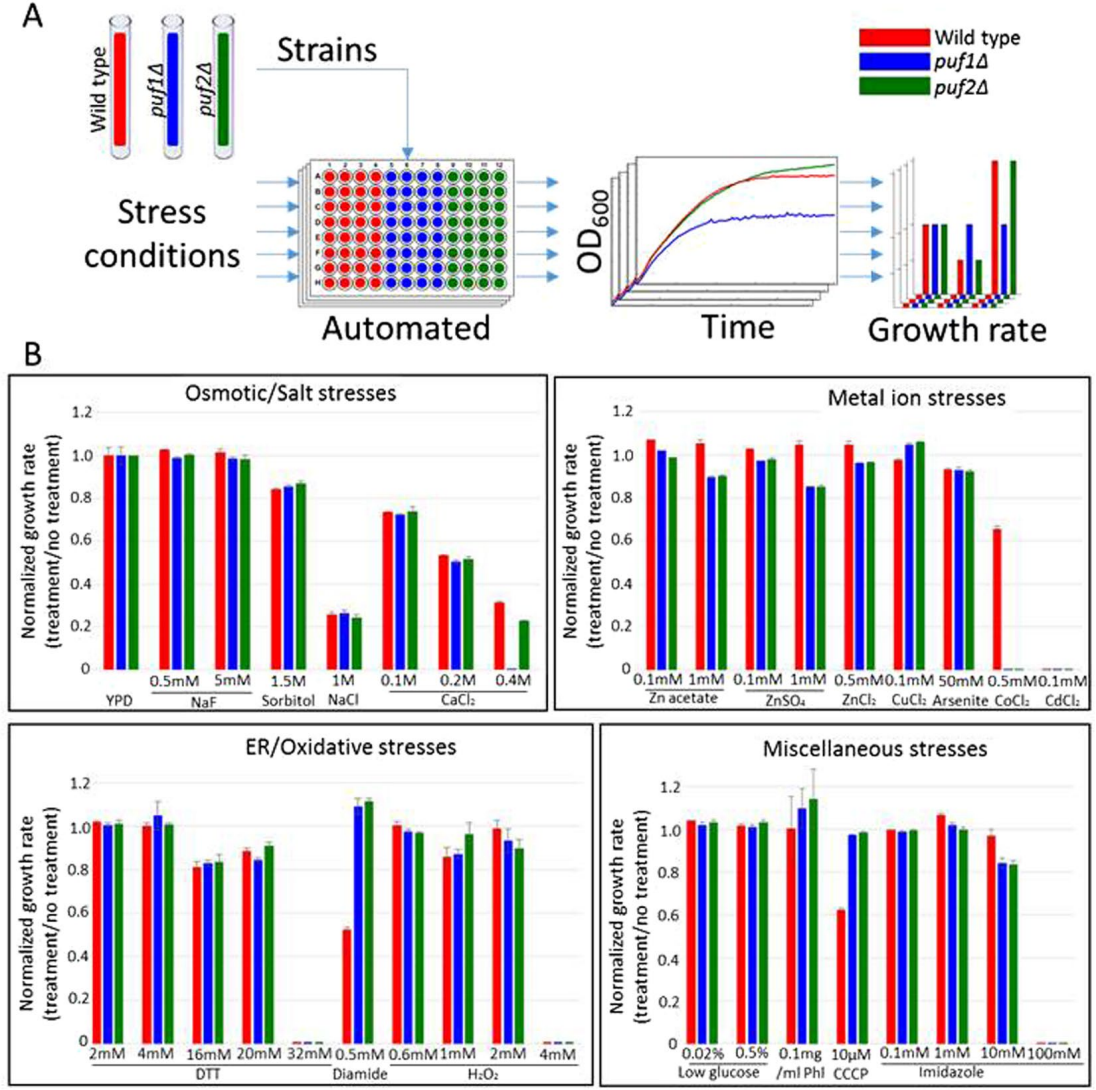
Stress conditions screen of *puf1Δ* and *puf2Δ* strains growth rate. (**A**) Scheme of the screen. Yeasts (yA635, yA636 and yA637) were grown in YPD to the mid- logarithmic phase and aliquoted to 96-wells plates containing media with the indicated supplements. Cells were grown for several hours, and OD_600_ was measured automatically every 15 min. Growth curves were acquired, and growth rates were calculated and normalized to untreated cells. (**B**) Representative stress conditions were divided into five general groups, and normalized growth rates were calculated for each stress condition. Error bars are +1 standard deviation, n ≥ 3. Phl-1,10-Phenantroline.

*puf1Δ* and *puf2Δ* strains show similar growth under standard conditions tested thus far. We therefore reasoned that their importance will be distinct under specific stress conditions. To this end, we performed an extensive screen for growth conditions in which *puf1Δ* or *puf2Δ* show altered growth compared to their isogenic parental strains. Strains were grown in 96-wells plates in liquid media with the indicated supplements (Fig. 1), and OD_600_ was measured every 15 min using an automated robotic system. All measurements were made at least in triplicate, and the doubling time during the logarithmic phase was determined (Fig. 1B). Considering the association of Puf1 and Puf2 with mRNAs encoding proteins of the cell periphery^13, 15^, we focused on growth conditions that would induce stress that is related to ER, plasma membrane or cell wall. Several additional conditions were tested on solid media (i.e., YPD agar) by the standard ‘drop test’. Overall, 86 growth conditions were tested (Supplementary Table 1). For each condition, the doubling time was normalized to growth in the same medium without an effector. This normalization was necessary to care for differences in growth between solid or liquid media, YPD or synthetic media, etc. Generally, no impact of Puf1 or Puf2 deletion was observed for the vast majority of conditions tested. Interestingly, both deletion strains grew better than their parental strains in the presence of 0.5 mM Diamide and in the presence of the ionic uncoupler CCCP. On the other hand, growth in the presence of 0.5 mM CoCl_2_ led to complete inhibition of *puf1Δ* and *puf2Δ* growth. A differential inhibitory effect was apparent only in the presence of CaCl_2_, whereby at 0.4 M, *puf1Δ* grew much slower than the parental strain and *puf2Δ*.

**Figure 2:**
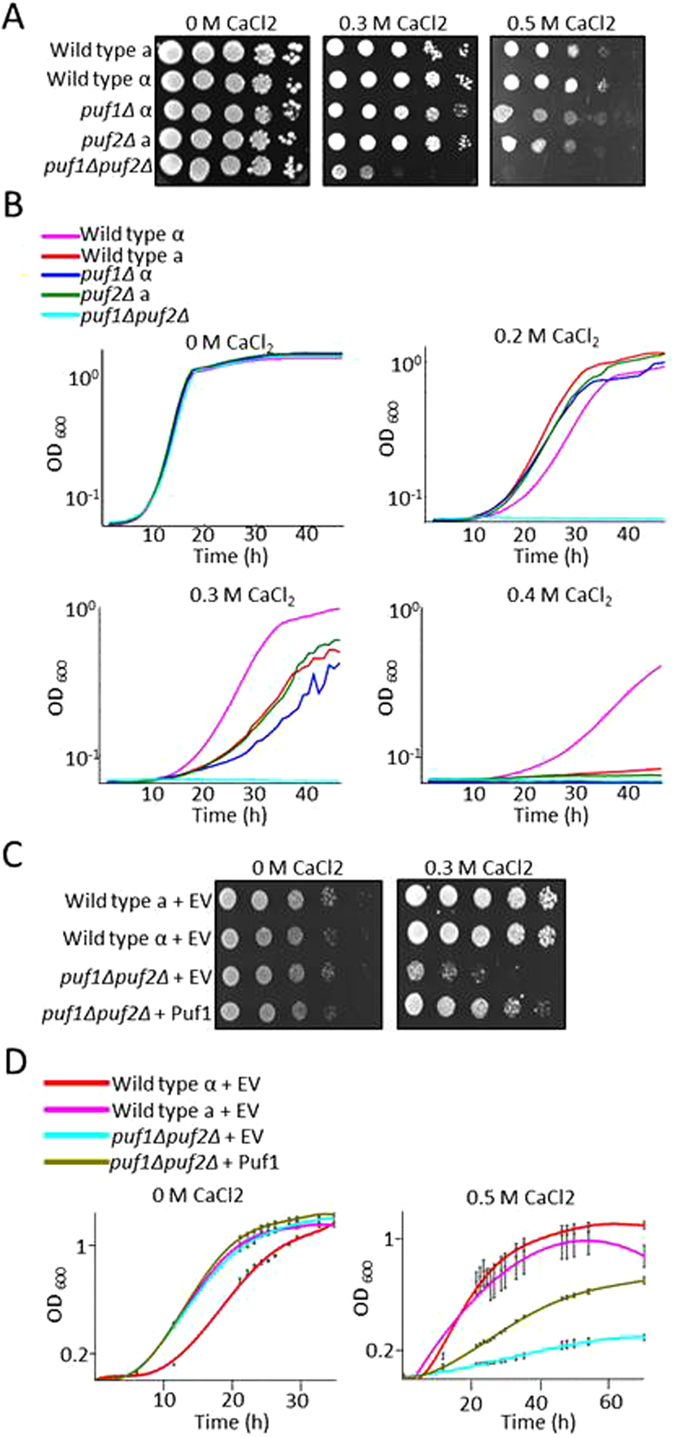
Puf1 and Puf2 deletions show distinct effects during growth under CaCl_2_ stress. (**A**) Cells were grown in YPD to the stationary phase and diluted to OD_600_ = 1. Five µl of this suspension and four subsequent 10-fold serial dilutions of wild-type mating type ‘a’ (WT a)(yA635), wild-type mating type α (WT α, yA995), *puf1Δ* (yA1382), *puf2Δ* (yA637) and *puf1Δpuf2Δ* (yA639) strains were spotted onto YPD agar with the indicated CaCl_2_ concentrations. Plates were incubated at 30 °C for one day without CaCl_2_ or three days with CaCl_2_. (**B**) Mid-log-phase cells were diluted to OD_600_ = 10^−3^, and 200 µl were aliquoted to 96-wells plates with the indicated CaCl_2_ concentrations in YPD. OD_600_ was measured automatically every 15 min. (**C**) WT ‘a’ with an empty vector (WT ‘a’ +EV (yA1306), WT α + EV (yA1419), *puf1Δpuf2Δ* + EV (yA1423) and *puf1Δpuf2Δ* with pPUF1 (yA1428), were spotted in 10-fold serial dilutions onto synthetic selection plates (SD) with the indicated CaCl_2_ concentrations. The strains were incubated at 30 °C for two days without CaCl_2_ or seven days with CaCl_2_. (**D**) Mid-log phase strains as in (**C**) were diluted to OD_600_ = 10^−2^; 200 µl of this suspension aliquoted to 96-wells plate with SD and proper selection media and the indicated CaCl_2_ concentrations. OD_600_ was measured at the indicated timepoints.

To substantiate the phenotypes on CaCl_2_, we assayed for growth on solid media (Fig. 2A). Herein we included strains that are of either mating type or a strain that is deleted of both Puf1 and Puf2. A clear synergistic effect is observed upon deletion of both genes. Synergism is also apparent when the strains are grown in liquid media and growth is measured continuously (Fig. 2B). Importantly, re-introducing a plasmid that expresses Puf1 from its native promoter significantly improves growth of the double deletion strain (Fig. 2C,D) and of *puf1Δ* (Supplementary Figure 1).

**Figure 3:**
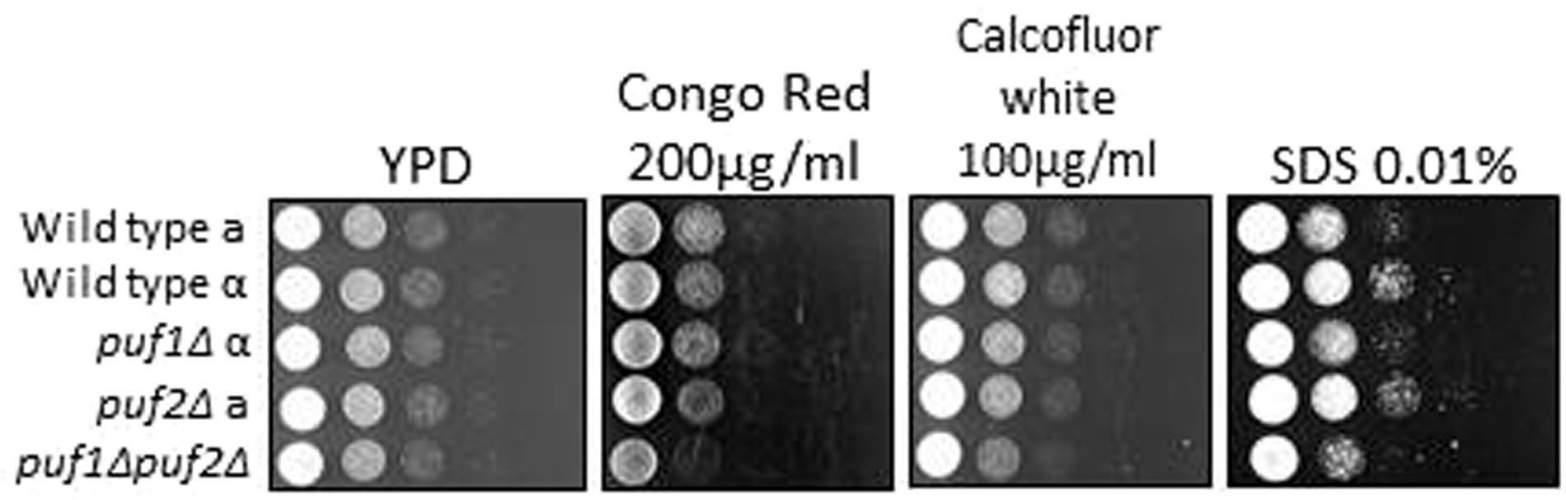
Effect of Puf1 and Puf2 deletions on growth under cell wall stress conditions. Wild-type mating type ‘a’ (WT ‘a’) (yA635), wild-type mating type α (WT α)(yA995), *puf1Δ* (yA1382), *puf2Δ* (yA637) and *puf1Δpuf2Δ* (yA639) strains were grown in YPD and plated on YPD agar with the indicated cell wall stressors in 10-fold serial dilutions. Plates were incubated at 30 °C for one day (YPD, Congo red, Calcofluor white) or three days (SDS).

CaCl_2_ at the concentrations used here may induce a strong osmotic stress that affects the plasma membrane and cell wall. We therefore tested the growth of these deletions under conditions that are known to induce cell wall and plasma membrane stress. Cells were grown on YPD plates supplemented with either 0.01% SDS, 0.1 mg/ml Calcofluor white (CFW) or Congo Red (CR). Marginal effects, if any, were observed with these agents (Fig. 3). This is consistent with the lack of effect when cells were subjected to cell wall stresses in liquid media (i.e., 1.5 M Sorbitol and 1 M NaCl shown in Fig. 1). Thus, a high CaCl_2_ concentration induces a unique effect.

### Transcriptome profiling

**Figure 4:**
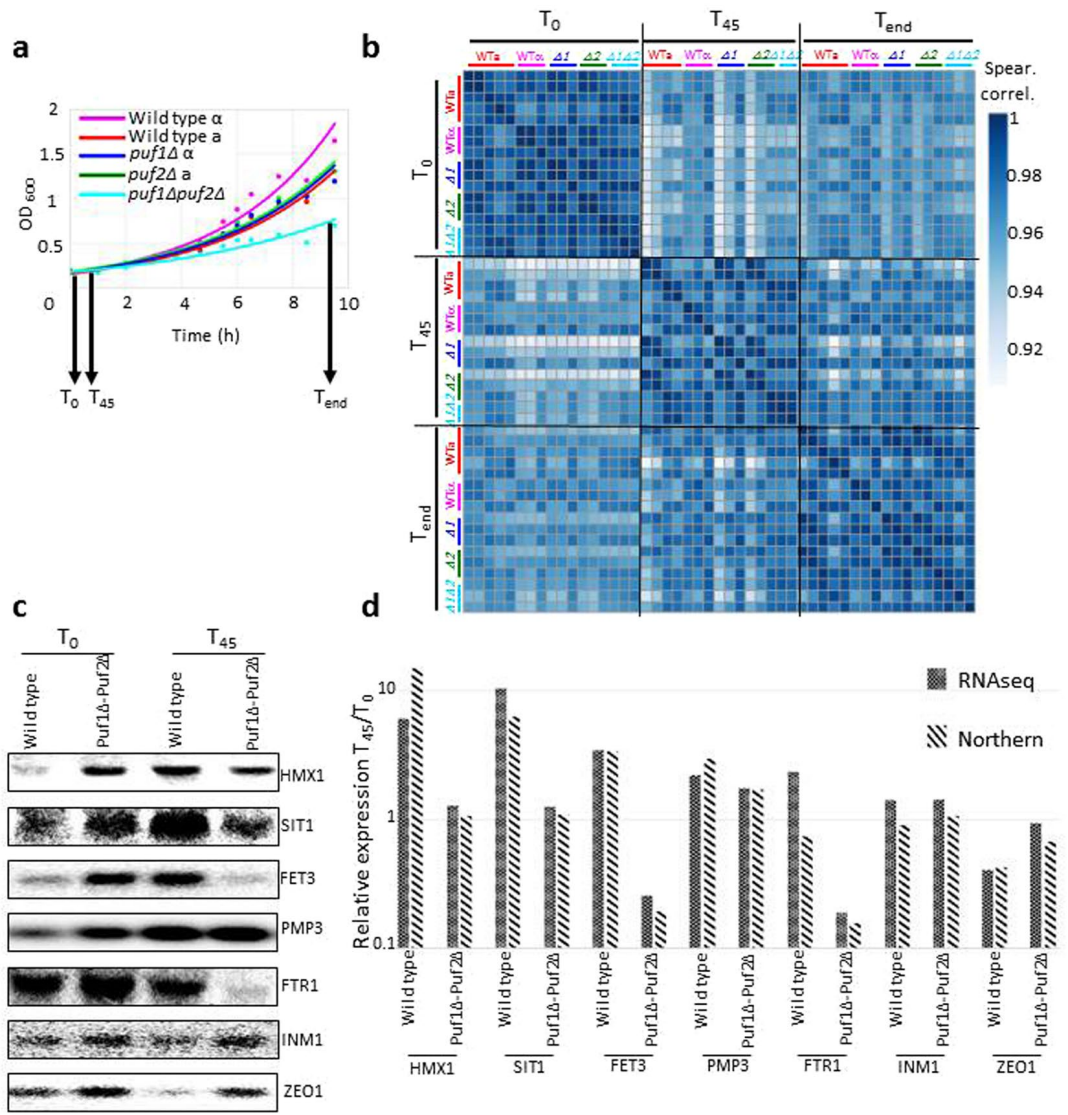
RNA-seq reproducibility and validation. (**a**) Growth curves of the five strains (yA635, yA995, yA1382, yA637, yA639) that were subjected to RNA-seq analysis. RNA was isolated before the addition of 0.3 M CaCl_2_ (T_0_), after 45 minutes of CaCl_2_ addition (T_45_), and after ~9 hours (T_end_). Samples were subjected to RNA-seq or northern analyses. (**b**) Spearman’s correlation heatmap of unclustered RNA-seq biological replicates for all strains at three timepoints. Note that the scale of the Spearman values starts at 0.9. Actual values are provided in Supplementary Table 2. (**c**) Northern analyses of total RNA extracted from wild-type (yA635) and *puf1Δpuf2Δ* (yA639) strains for untreated (T_0_) and 0.3 M CaCl_2_ treated (T_45_) samples. Radiolabeled PCR products of the genes indicated to the right were used as probes. (**d**) Bands in the northern blot (**c**) were quantified with ImageQuant, and the ratio for the signals at T_45_ to T_0_ are presented for each gene (stripes). RNA-seq results (weave) for the same genes from the same biological replica are presented. Compatibility of the results was calculated using Pearson correlation (*p value* = 0.005).

To identify genes that are affected by CaCl_2_ addition and to define the differential phenotype of Puf1 and Puf2, we performed RNA-seq analyses. *puf1Δ* and its parental strain, *puf2Δ* and its parental and *puf1Δpuf2Δ* strain were grown in YPD supplemented with 0.3 M CaCl_2_ (a sub-lethal concentration for the strains when grown in YPD). Aliquots were collected before the addition of CaCl_2_ (T_0_), 45 min after the addition (T_45_) and after ~9 hr (T_end_) (Fig. 4a). At least three independent biological replicates were performed for each strain at each timepoint, except for *puf1Δpuf2Δ* at T_45_ with only two replicates. Each replicate entailed independent cell growth, RNA extraction and RNA-seq analysis in order to account for both biological and technical variations. Thus, we had multiple measurements from which we could evaluate the reproducibility of the results. For only five out of 49 pairwise comparisons of biological replicates, the Spearman rank correlation was below 0.95 and only one was below 0.9 (Fig. 4b, Supplementary Table 2). For each sample, we obtained more than 1 × 10^7^ reads that were mapped to 6,600 yeast genes (Supplementary Table 2). At least 75% of the genes had more than 100 reads, indicative of excellent coverage. To maintain consistency, for all further analyses we selected only genes that had more than 100 reads in at least one sample, yielding a background list of 5,034 genes.

To validate the RNA-seq results using an alternative approach, we measured the changes in expression by northern analysis. RNA samples were collected from the five strains (WTa, WTα, *puf1Δ*, *puf2Δ*, *pu1Δpuf2Δ*) at three timepoints (T_0_, T_45_ and T_end_) and tested with probes for seven different genes representing a large range of effects (up to a 10-fold change). The gene INM1 represents a gene with no change in both the WT and deletion strains. Expression changes measured by northern analysis were then compared to those measured by RNA-seq. Figure 4c,d present a sample of these comparisons (using the same RNA samples) for the WT and *puf1Δpuf2Δ* cells at T_45_. Overall, 36 comparisons were performed and highly similar trends were obtained with a *p value* < 10^−6^ (Supplementary Figure 2). These data, obtained by an alternative method, support the validity of the RNA-seq data.

### Impact of CaCl_2_ on gene expression

**Figure 5:**
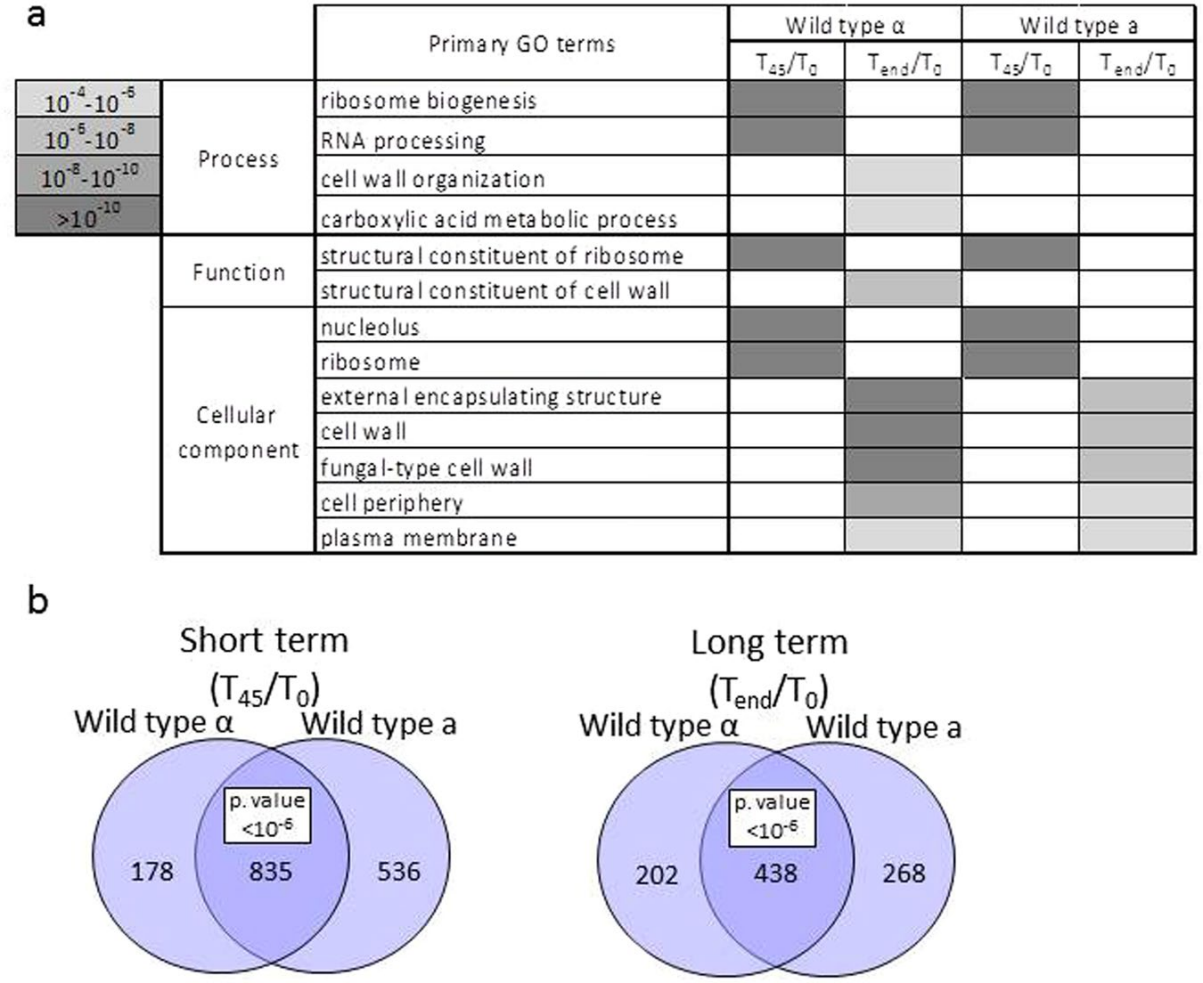
Transcriptomic changes imposed by CaCl_2_ on wild-type strains. (**a**) Heatmap of SGD GO enrichment analysis of significantly affected genes in T45/T0 and Tend/T0 in the two wild-type strains. The grey shades indicate levels of enrichment score of each GO term. Only primary GO terms with p value < 10e−4 and FDR ≤ 0.01 are presented. Full results are presented in Supplementary Table S3. (**b**) Venn diagram of significantly affected genes (changed by more than two fold relative to T0, adjusted p value 0.05) in WT ‘a’ (yA635) and WT α (yA995). The hypergeometric test was used to calculate significance for the merged group.

The transcriptomic response of yeast cells to high CaCl_2_ treatment was examined previously with DNA microarrays^33^. To expand these data, we first analyzed the expression changes that occur for the parental strains (one of each mating type). For each gene, we determined its fold change at T_45_ (immediate response) and T_end_ (long-term exposure) (Supplementary Table 3). We applied DESeq2 to identify genes that were affected by at least two-fold (either induced or repressed in each timepoint) (Supplementary Table 3). Gene Ontology (GO) enrichment analysis at the Saccharomyces Genome Database (SGD) site revealed a significant enrichment for genes encoding constituents of ribosomes (i.e. many ribosomal proteins or ribosome-associated proteins). Interestingly, genes encoding proteins that are involved in rRNA metabolism (e.g., ribosome biogenesis and rRNA processing) are strongly affected at the immediate response. Within the long-term response, gene ontologies that are related to the cell wall are enriched. Such a response is expected following the osmotic and ionic changes that occur in the media. The overall response to CaCl_2_ is similar for the two mating types, and similar GO terms are enriched in the two mating types (Fig. 5a). Furthermore, a significant overlap is apparent between the lists of genes that are affected in the two strains (Fig. 5b) (Supplementary Table 3).

### Impact of Puf1 and Puf2 deletions on gene expression in CaCl_2_

To identify changes due to PUF deletion, we compared the response to CaCl_2_ of each deletion strain with the response of its corresponding parental strain. We initially exploited the same bioinformatics approach as above (i.e. employing DESeq2 fold change cutoff of two fold). However, very few genes were assigned with an acceptable adjusted *p* value by this approach (Supplementary Table 4). This might be because the expression changes are small among our samples and/or variation between repeats is high. We note that our three biological replicates were totally independent ones, whereby colonies were picked from different plates (streak from frozen stocks) and grown on different days (with a different batch of medium); RNA was extracted on different days (usually with a different batch of reagents); libraries were prepared and sequenced on different days (in some cases on different machines). Variation is relatively high with such a set-up, and use of fold changes statistics with DESeq2 will probably necessitate many more repeats to derive significant values. Recently, Schurch *et al*.^34^, while utilizing a much more conservative experimental set-up than ours, suggested that at least 6 repeats are necessary for DESeq2 to define statistically significant genes with two fold changes. Thus, when true biological repeats are done (as herein), an even higher number of repeats is probably necessary to define genes as significantly changing by more than two fold.

**Figure 6:**
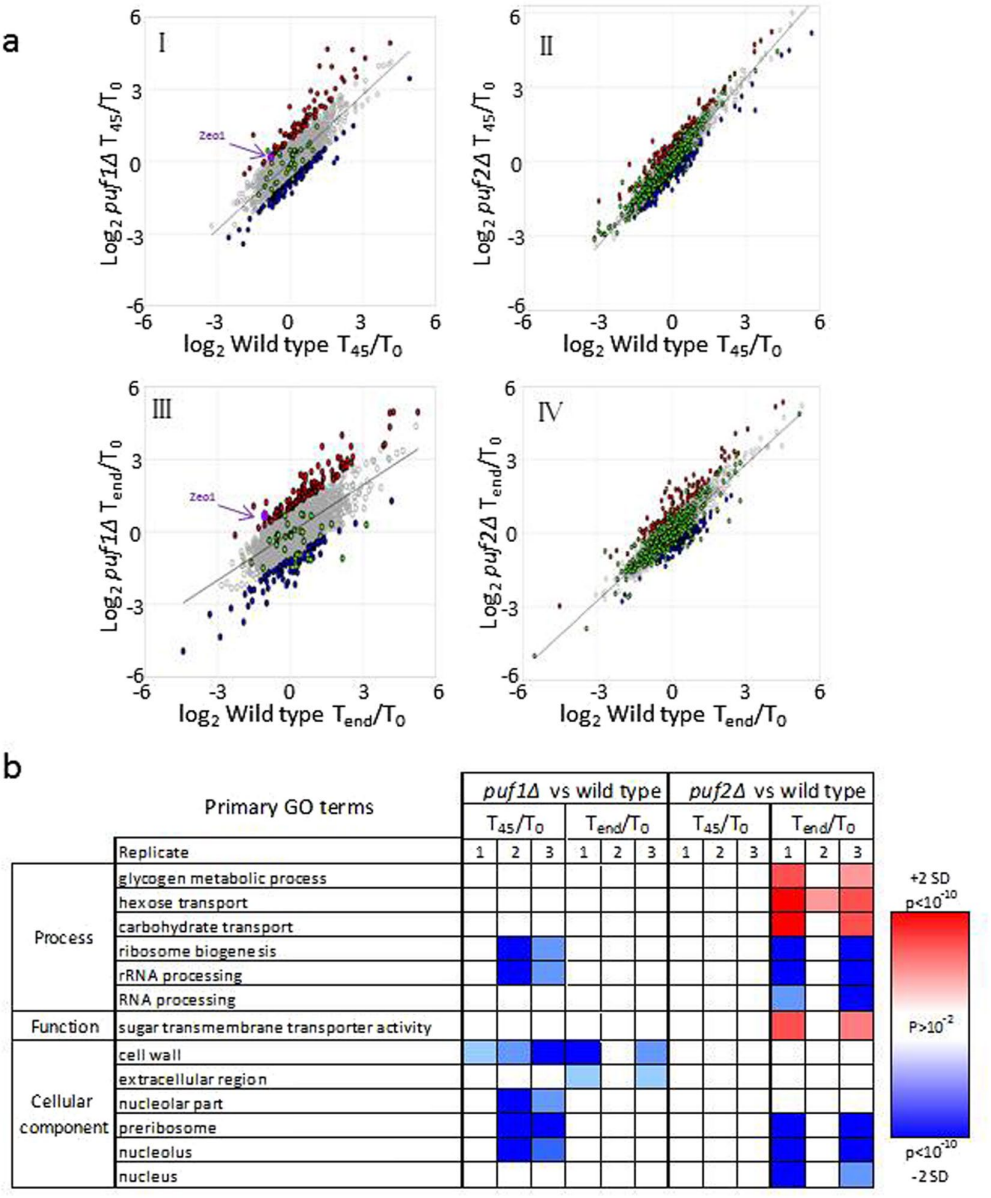
Distinct effects of Puf1 and Puf2 on gene expression. (**a**) Scatterplots of log_2_ normalized reads of *puf1Δ* (yA1382) against its isogenic wild-type (yA995) in T_45_/T_0_ (I) and T_end_/T_0_ (II), and *puf2Δ* (yA637) against its isogenic wild-type (yA635) in T_45_/T_0_ (III) and T_end_/T_0_ (IV). Genes significantly affected from the knockouts are colored in red (significantly higher effect) or in blue (significantly lower effect). Genes that were previously shown to be bound by Puf1^13^ or Puf2^13, 15^ are indicated in green. This scatter is for one of the biological replicates, and the additional ones are shown in Supplementary Table S4. (**b**) Heatmap of SGD GO enrichment analysis of significantly affected genes in T_45_/T_0_ and T_end_/T_0_ in *puf1∆* and *puf2∆* relative to their isogenic wild-type strains. The red or blue shades indicate levels of enrichment score if induced or repressed, respectively. The color intensity indicates the level of *p* value enrichment score of each GO term. Only primary GO terms with p value <10e^−2^ and FDR < 10^−2^ were included. Full results are presented in Supplementary Table S4.

We therefore applied an alternative approach, in which we selected as most affected genes those that deviate by more than two standard deviations (SD) from the average change for each timepoint. Normally, ~2.5% of the genes (i.e., ~100 genes) show an effect that is higher than 2 SD from the average response (i.e., induced genes) and a similar number are repressed by >2 SD (Fig. 6). The use of selection criteria that is based on deviation from the average response avoids the need to set an arbitrary cutoff of fold change. In order not to introduce variation from one repeat to another, each experiment was treated separately. Figure 6a presents scatter plots of the changes for both deletions at the two timepoints from one of the biological replicates (the data for the two others are presented in Supplementary Table S4). The scatter of the changes in *puf1Δ* is clearly larger than the scatter of *puf2Δ* at T_45_ and T_end_ (Fig. 6a, panels *I* and *III* vs *II* and *IV*), i.e., the expression changes in *puf2Δ* are much milder that those of *puf1Δ*. These differences in expression are consistent with the stronger impact on growth of *puf1Δ* elicited by CaCl_2_ (Fig. 2).

For each timepoint, we identified genes that deviate the most from the common transcriptomic change (i.e., deviated by more than 2 SD from the best-fit linear trend line) (Fig. 6a). Those with relatively increased expression are marked in blue and those with relatively lower expression are marked in red (Fig. 6a) (gene lists are shown in Supplementary Table S4). GO term analyses were performed for each of the three biological repeats, and terms that appeared in two or more repeats are presented in Fig. 6b (all GO term data are presented in Supplementary Table 4). Significant differences are observed in the responses of the deletions. Most notable is the effect on cell periphery components that appears only for *puf1Δ* consistent with its severe growth phenotype. On the other hand, genes that are important for carbohydrate transport appear to be induced only in the *puf2Δ* at its long-term response. Genes with roles in ribosome biogenesis appear to be repressed in both strains, yet while the effect for *puf1Δ* is in the short term, for *puf2Δ* it occurs at T_end_ (Fig. 6b). Although these data are corrected for changes in the parental strains, we note that this group of genes is also affected in the parental strains (Fig. 5b); it should therefore be taken into account cautiously. Nevertheless, PUF proteins were linked recently to ribosome biogenesis due to localization to the nucleolus, binding to rRNA or regulation of factors involved in synthesis^35–37^.

The repertoire of mRNAs that are physically associated with Puf1 or Puf2 was determined previously^13, 15^. These genes are indicated by green dots in Fig. 6a (and in Supplementary Table S4). We tested whether this group of genes is affected preferentially from the deletion of their corresponding Puf. Statistical analysis revealed enrichment only for mRNAs that are bound by Puf1: at T_45_, eight of the 38 mRNAs known to be bound by Puf1^13^ appeared to have a relatively lower change upon Puf1 deletion (reside among the genes labeled blue) (*p* value of 8e^−6^, hypergeometric test). Interestingly, only one mRNA (*ZEO1*) showed relatively higher amounts at T_45_. Of the 501 mRNAs that were found to be associated with Puf2^13, 15^, only 32 showed a significant change upon this protein deletion. This number is statistically insignificant (*p* value 0.02, hypergeometric test). We performed a bioinformatics analysis to determine whether the affected mRNAs contain the RNA sequence that is recognized by Puf2 (dual UAAU motif)^17^. No enrichment for this motif was found using the AME tool of the MEME suit^38^ or the DRIMust tool^39^. Taken together, our analyses reveal that already after 45 min, the expression of mRNAs that were not known to be bound by Puf1 or Puf2 changes. This may suggest that downstream effects accumulate quickly after application of CaCl_2_, thereby obscuring the direct impact conferred by these proteins on their targets. Alternatively, RNA-binding domains outside the PUM domain may play a dominant role under this condition; notably, Puf1 and Puf2 contain a putative RRM motif in their N-terminal half. In summary, although under optimal media a high similarity exists between Puf1 and Puf2 in their binding motif and bound mRNAs^13, 17^, a significant difference in expression pattern is apparent under stress conditions upon their deletion. Thus, while seemingly redundant, these proteins may execute different functions by yet-to-be determined mechanisms.

### Impact of the double deletion on gene expression

**Figure 7:**
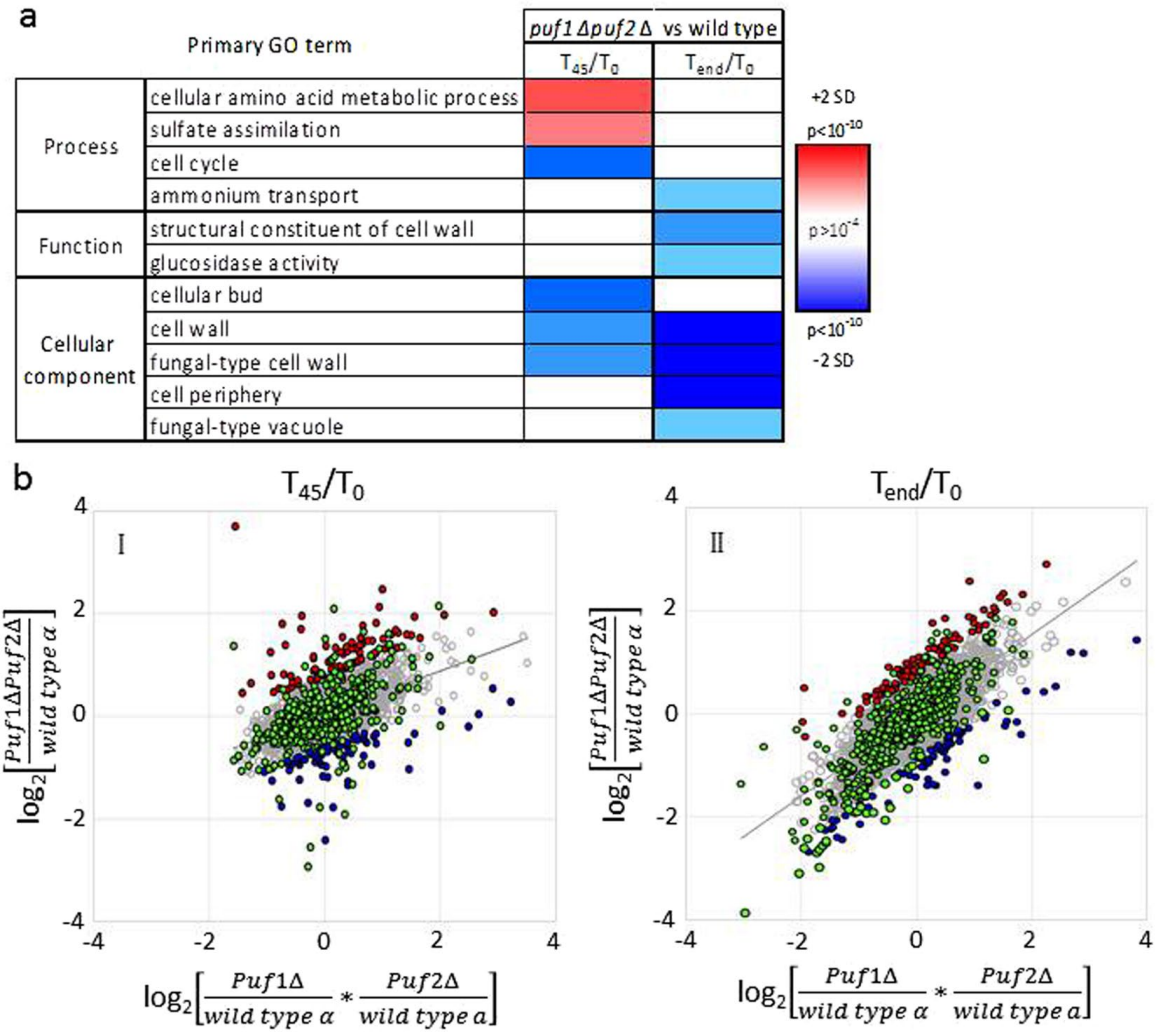
Effect of Puf1 and Puf2 double deletion. (**a**) Heatmap of SGD GO enrichment analysis of significantly affected genes in T_45_/T_0_ and T_end_/T_0_ in *puf1∆puf2∆* relative to wild-type α. The red or blue shades indicate levels of enrichment score of induced or repressed respectively, and the color intensity indicates the level of *p* value enrichment score of each GO term. Only primary GO terms with *p value* < 10e^−4^ and FDR ≤ 0.01 are presented. (**b**) Scatterplots of log_2_ normalized reads of *puf1Δpuf2Δ*/WT α against multiplication of *puf1Δ*/WT α and *puf2Δ*/WT ‘a’ in T_45_/T_0_ (I) and T_end_/T_0_ (II). Genes significantly affected by the synergism of Puf1 and Puf2 knockouts are colored in red (two standard deviations above the trend line) or in blue (two standard deviations below the trend line). Genes bound by Puf1 or Puf2^13, 15^ within the two standard deviations areas are colored in green.

The expression changes within the double knockout were measured and compared to the WT parental strain. Unfortunately, since at the short term (T_45_) we had only two experimental repeats, no gene was assigned by DESeq2 as changing by more than two fold at a significant level (adjusted p value < 0.05) (Supplementary Table 5). Furthermore, at the long term response (T_end_), only 24 genes were assigned as changing by more than two fold (either induced or repressed), relative to T_0_ with an adjusted p value < 0.05 (Supplementary Table 5). This small number is probably due to the aforementioned batch effect that is associated with our biological repeats which leads to filtering of many genes by DESeq2. We therefore performed GO term analysis for one of the experimental repeats, for which we had a complete set of data from the same batch (Fig. 7a). While some expected ontologies change (cell wall, cell cycle, cell periphery), other like sulfate assimilation are more intriguing to explain.

The exacerbated growth defect of the double deletion strain compared to the single deletions suggests a cooperative function in some target genes. With the aim of finding genes that are affected to a much greater extent in the double knockout, we compared the observed expression changes of *puf1Δpuf2Δ* to the expected change from the single mutants effects (the product of the expression changes for each mutant compared to its parental strain) (Supplementary Table 5). Figure 7b presents a comparison of the changes after 45 min (left) and after long exposure (right) for data from one of the biological replicates. Genes that show a significantly higher expression (>2 SD) in the double deletion compared to that expected from the product of the single deletions are presented as red dots, and those with a significantly lower expression are presented as blue dots. These genes are likely to be regulated in a coordinated manner by both proteins. Genes that were shown to be associated physically with Puf1 or Puf2 are enriched significantly among these groups (Fig. 7b, green dots, *p* < 3.5 × 10^−4^ in either case). Thus, mRNAs associated physically with Puf1 or Puf2 proteins appear to be affected much more significantly upon their double deletion. It appears that while some mRNAs increase their relative abundance (i.e., are in cohort with the red dots), others show decreased abundance (i.e., are in cohort with the blue dots). This indicates that Puf1 and Puf2 exert different expression impacts on different mRNAs.

### Role for *ZEO1* in mediating Puf1p and Puf2p impact

**Figure 8:**
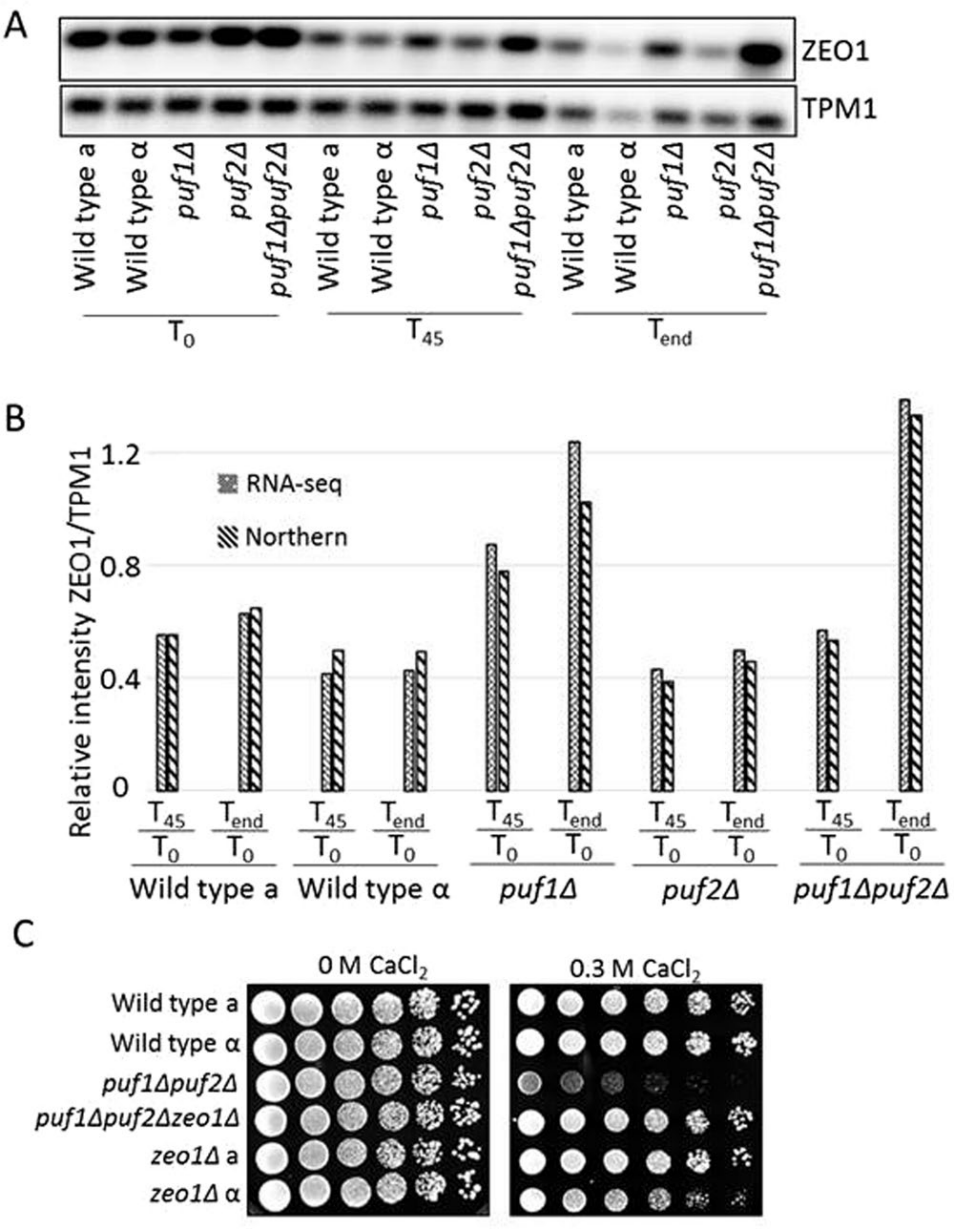
Effect of *ZEO1* deletion on cell growth under CaCl_2_ stress. (**A**) Total RNA was extracted from the indicated strains at the indicated times and subjected to northern analysis with probes for *Zeo1* and *TPM1* as a control. (**B**) Bar diagram shows the ratio of *ZEO1* versus *TPM1* transcripts from quantification either by northern blot or RNA-seq of expression in the same biological replicate. (**C**) Cells from the indicated strains were diluted to OD_600_ 1.0, and five-fold serial dilutions were spotted onto YPD agar with the indicated CaCl_2_ concentrations and were incubated at 30 °C for two days without CaCl_2_ or three days with CaCl_2_.

We next sought to focus on candidate genes that might mediate the slow growth effect of the Puf knockouts under calcium stress. *ZEO1* encodes a protein that is associated with the plasma membrane and is involved in the response to cell wall stress^40^. The mRNA of Zeo1 was shown previously to be bound by Puf1p and Puf2p^13^. Northern analysis revealed that *ZEO1* mRNA levels are maintained higher in *puf1Δ* and *puf1Δpuf2Δ* upon CaCl_2_ treatment, and do not decrease as in the WT or *puf2Δ* strains (Fig. 8A,B). This correlates with the growth phenotype of the different strains.

The expression data suggest that Puf1p maintains low levels of *ZEO1* mRNA, which permit growth at high CaCl_2_ concentrations. To test whether low levels of Zeo1 can restore the mis-regulation that is conferred upon Puf1 and Puf2 deletion, we deleted the *ZEO1* gene from *puf1Δpuf2Δ*. The triple deletion strain was grown in the presence of 0.3 M CaCl_2_ (Fig. 8C), and was found to completely restore the growth defect of *puf1Δpuf2Δ*. This indicates that *ZEO1* functions downstream to *PUF1*, and its presence is critical for the growth defect of *puf1Δpuf2Δ*.

## Discussion

In this work, we identified growth conditions in which Puf1 and Puf2 appear to be critical. In only four of the 86 conditions did the two deletion strains grow slower than their parental strain and only upon one stress (high CaCl_2_ concentration), an apparent effect that differs between the two deletion strains. Having a narrow scope of conditions under which the role of an RBP is essential is not surprising as these proteins are considered to exert a post-transcriptional regulatory role by fine-tuning the activity of several target genes. For example, the decay rates of only three out of 21 target mRNAs were affected upon deletion of Puf protein when examined during the logarithmic growth phase^21^. This mode of activity is consistent with the involvement of RBPs under very specific stress conditions. Indeed, Russo and Olivas have shown a differential regulation of the Puf target YHB1 in different carbon sources or culture densities^23^. Nevertheless, considering the list of mRNAs that are bound by Puf1 and Puf2, we expected a somewhat broader effect that would include other conditions affecting the cell periphery. For example, growth under high NaCl concentrations, which elicit several cell periphery related effects, did not appear to be dependent on either Puf1 or Puf2. High NaCl is of particular interest also because it is known to activate the calcineurin pathway, similarly to CaCl_2_^33^. A comparative analysis of the responses to high CaCl_2_ and high NaCl revealed more than 100 genes that are affected only by the former^33^. These may be connected to Puf proteins and hence elicit a CaCl_2_-specific effect.

The partial growth defect of *puf1Δ* indicates that Puf2 cannot fully compensate for the absence of Puf1. This may be because there are genes that Puf1 regulate (presumably by binding their mRNA) and Puf2 does not. While few Puf1-specific targets were suggested previously (NOP1, SSN2, YGL034C and CDC27)^13^, none have an obvious relevance to CaCl_2_ stress. Importantly, we observed a significant mismatch between the list of mRNAs whose expression is affected under CaCl_2_ stress and the list of mRNAs found previously to be bound by these proteins^13^. This may suggest that under CaCl_2_ stress, Puf1 and Puf2 bind a different repertoire of mRNAs than when grown in an optimal medium. We were unable to obtain adequate RNA immunoprecipitation under high CaCl_2_, most likely due to the release of Ca-dependent proteases and RNases under these conditions. This hypothesis is therefore yet to be confirmed.

The results herein reveal a stress condition in which Puf1 is important for optimal growth. Puf2 is partially compensating for the loss of Puf1 as a much stronger effect is apparent upon deletion of both Puf1 and Puf2. Puf1 and Puf2 bind a similar target sequence^17^ and almost all mRNAs bound by Puf1 are also bound by Puf2^13^. This suggests that redundancy is brought about through overlapping activities on the same target mRNAs. This overlap maintains proper expression of target transcripts in the absence of either Puf1 or Puf2. By studying a strain deleted of both Puf1 and Puf2, we were able to identify a group of mRNAs that are affected in the double deletion to a much greater extent than in the sum of the single mutants (Fig. 7). This group was enriched with genes that were shown to be bound by Puf1 or Puf2^13, 15^. Such enrichment was much weaker for the single deletions, thus suggesting a synergistic association between the two. Targets for Puf1 and Puf2 were found previously to contain a dual UAAU motif, i.e., two UAAU sequences separated by a short linker, which can be bound also by Puf1^17^. It was found recently that a single Puf2 protein can bind a single UAAU^15^, thus suggesting a dimerization on UAAU motifs. We noticed that among the genes most affected in the double deletion strain, there are relatively more genes with two or more UAAU tetranucleotides compared to the entire genome (*p* value of 0.002, hypergeometric test). It may therefore be that the synergy between Puf1 and Puf2 is achieved through their heterodimerization on their targets.

Zeo1 protein is associated with the plasma membrane and affects the cell wall integrity pathway^40^. This pathway is important for proper response to insults to the cell wall. While in WT and *puf2Δ* the mRNA levels of *ZEO1* decreased upon CaCl_2_ treatment, they failed to decrease in *puf1Δ* and the double deletion (Fig. 8). This correlates with the growth defect of *puf1Δ* and *puf1Δpuf2Δ*. Furthermore, *ZEO1* mRNA is bound by Puf1p and Puf2p, and its 3′UTR includes multiple UAAU motifs. This suggests a regulatory pathway in which Puf1p and to a lower extent Puf2p bind *ZEO1* mRNA and maintain low levels of the protein upon CaCl_2_ addition. The importance of having low levels of Zeo1p to maintain growth upon CaCl_2_ stress is also apparent from the restoration of growth of the triple deletion strain.

**Figure 9:**
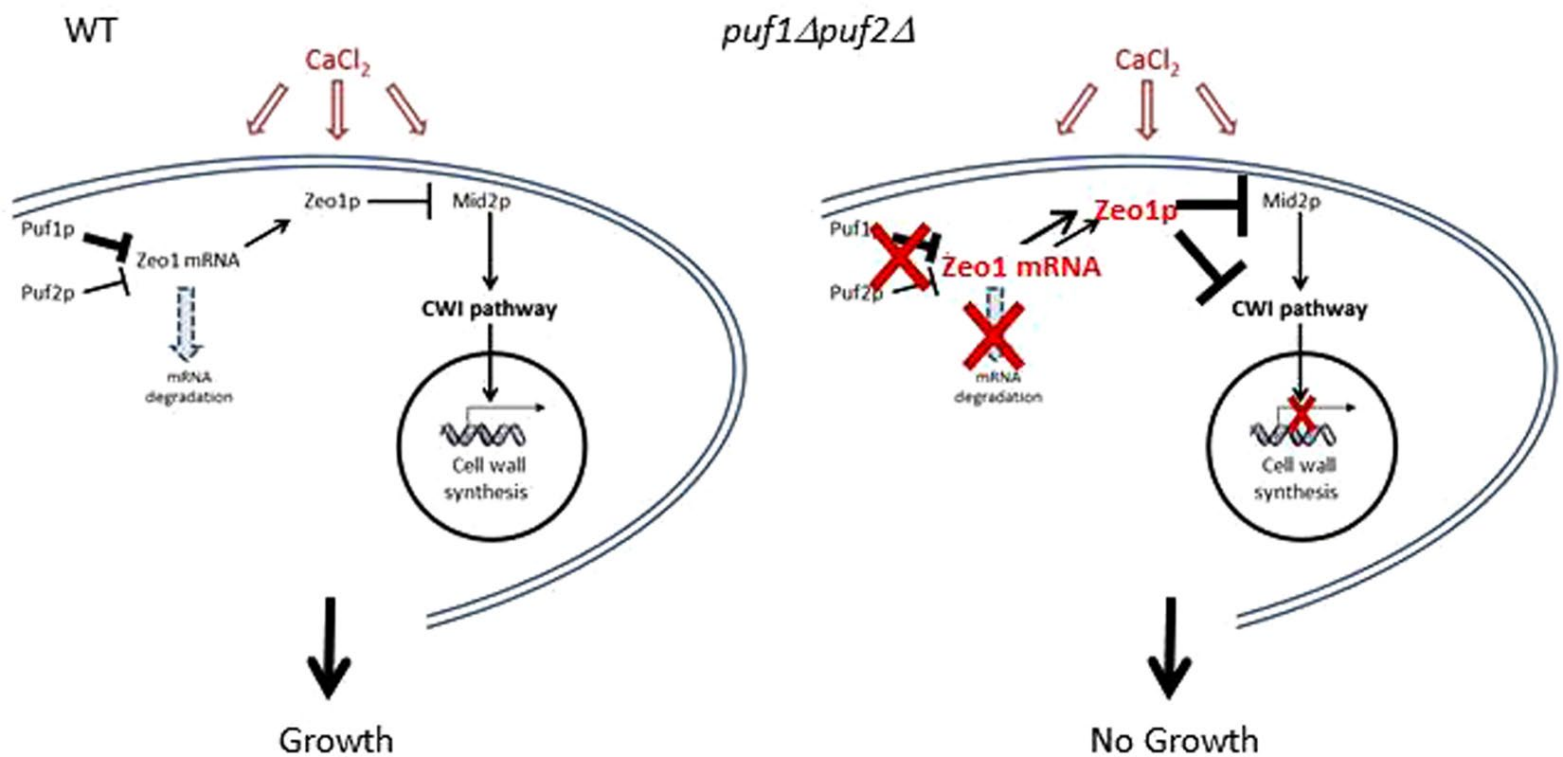
Model for cellular regulation upon CaCl_2_ stress. High concentration of CaCl_2_ activates Puf1p and Puf2p proteins. These maintain low levels of Zeo1 mRNA, most likely by inducing its degradation. Thus, Zeo1 protein levels are low and its known repressive effect on the Cell Wall Integrity (CWI) pathway^40^ is excluded. This permits proper transcriptional response to the stress. In strains deleted of Puf1p and Puf2p (right panel), Zeo1 mRNA levels are high, hence an increase in Zeo1 protein levels and inhibition of the CWI pathway. This leads to improper transcriptional response and the observed growth defect.

We propose the following model for regulation upon CaCl_2_ stress (Fig. 9, left): CaCl_2_ stress activates Puf1p and Puf2p, and these maintain low levels of Zeo1 mRNA and hence low levels of Zeo1 protein. Considering the post-transcriptional roles of Puf proteins and the increased mRNA levels of Zeo1 in *puf1Δpuf2Δ*, we suggest that this occurs through degradation regulation. Since Zeo1p is a negative regulator of the Cell Wall Integrity (CWI) pathway^40^, its low levels are important for proper response to stress. In the knockout strains (Fig. 9, right), the increased Zeo1 mRNA levels (presumably due to reduced degradation) lead to an increase in Zeo1 protein. This probably inhibits the CWI pathway, thereby induce improper transcriptional response and a growth defect in CaCl_2_.

In conclusion, we identified growth conditions in which distinct physiological impact are apparent for proteins that were considered redundant. Consistent with the phenotypic difference, significant expression differences were apparent. Many of the affected genes did not have the established binding site of Puf1 and Puf2, suggesting either an indirect impact on these targets or binding to a novel binding site. Nevertheless, a synergistic effect was observed on many genes, suggesting an interaction between Puf1 and Puf2. This work expands our understanding about the impact of mRBPs on cellular physiology and underscores the multiplex nature of their action.

## Methods

### Yeast strains, plasmids and growth conditions

The *S*. *cerevisiae* strains used in this study are summarized in Table 1. WT ‘a’ (yA635). *puf1Δ* (yA636) and *puf2Δ* (yA637) were used for the stress conditions screen (Fig. 1) (same mating type), whereas for the transcriptome analysis (yA635) (yA995) (yA1382) (yA637) (yA639) were used. For the rescue analysis, PCR-based methods generated pPuf1-FLAG with its original promoter and 3′ UTR in YCpLac22^41^. Knockout of *ZEO1* ORF was achieved using direct replacement with TRP1^42^, and was verified with PCR and northern analysis. Sequencing found one mutation I727T, and expression was verified using western analysis^43^. Cells were grown either in YPD (1% yeast extract, 2% peptone and 2% glucose) or SCD medium (0.17% yeast nitrogen base, 0.5% ammonium sulfate, 2% glucose and appropriate amino acids) at 30 °C at 200 rpm. For CaCl_2_ addition, YPD had to be titrated to pH 5.5 (with 37% hydrochloric acid), and SCD was depleted of ammonium sulfate. No difference in growth was observed for the modified YPD or SCD. For large volume growth, cells were diluted to OD ~0.1 in 100–150 ml YPD pH 5.5 and grown at 30 °C at 200 rpm. For growth on solid media, saturated overnight cultures of each strain were diluted in sterile water to OD_600_ of 1 and used to make serial dilutions.

**Table 1:** Yeast strains used in this study.

Lab name	Deletion	Relevant genotype	Source
yA635	Wild-type	MAT**a**, *his4*-*539*, *leu2*-*3*,*112*, *trp1*-*1*, *ura3*-*52*, *cup1*::*LEU2/PM* (*PGK1pG/MFA2pG*)	29
yA995	Wild-type	MATα, *leu2*-*3*,*112*, *lys2*-201, *trp1*-*1*, *ura3*-*52*, *cup1*::*LEU2/PM*	29
yA636	*puf1Δ*	MAT**a**, *his4*-*539*, *leu2*-*3*,*112*, *trp1*-*1*, *ura3*-*52*, *cup1*::*LEU2/PM*, *puf1*::*Neo* ^r^	29
yA1382	*puf1Δ*	MATα, *leu2*-*3*,*112*, *lys2*-201, *trp1*-*1*, *ura3*-*52*, *cup1*::*LEU2/PM puf1*::*Neo* ^r^	29
yA637	*puf2Δ*	MAT**a**, *his4*-*539*, *leu2*-*3*,*112*, *trp1*-*1*, *ura3*-*52*, *cup1*::*LEU2/PM*, *puf2*::*URA3*	29
yA639	*puf1Δ*, *puf2Δ*	MATα, *leu2*-*3*,*112*, *lys2*, *trp1*-*1*, *ura3*-*52*, *cup1*::*LEU2/PM*, *puf1*::*Neo* ^r^, *puf2*::*URA3*	29
yA1306	Wild-type +EV	MAT**a**, *his4*-*539*, *leu2*-*3*,*112*, *trp1*-*1*, *ura3*-*52*, *cup1*::*LEU2/PM* (*PGK1pG/MFA2pG*), +pRS416 (CEN/URA3)	This study
yA1307	*puf1Δ*+*EV*	MAT**a**, *his4*-*539*, *leu2*-*3*,*112*, *trp1*-*1*, *ura3*-*52*, *cup1*::*LEU2/PM*, *puf1*::*Neo* ^r^, +pRS416 (CEN/URA3)	This study
yA1308	*puf1Δ*+*pPUF1*	MAT**a**, *his4*-*539*, *leu2*-*3*,*112*, *trp1*-*1*, *ura3*-*52*, *cup1*::*LEU2/PM*, *puf1*::*Neo* ^r^, pPUF1 [PUF1promoter-PUF1 ORF – HA –PUF1 3UTR in pRS416]	This study
yA1419	Wild-type +EV	MAT**a**, *his4*-*539*, *leu2*-*3*,*112*, *trp1*-*1*, *ura3*-*52*, *cup1*::*LEU2/PM* (*PGK1pG/MFA2pG*) + YCpLac22 (CEN/TRP1)	This study
yA1420	Wild-type +EV	MATα, *leu2*-*3*,*112*, *lys2*-201, *trp1*-*1*, *ura3*-*52*, *cup1*::*LEU2/PM*, +YCpLac22 (CEN/TRP1)	This study
yA1423	*puf1Δpuf2Δ*+*EV*	MATα, *leu2*-*3*,*112*, *lys2*, *trp1*-*1*, *ura3*-*52*, *cup1*::*LEU2/PM*, *puf1*::*Neo* ^r^, *puf2*::*URA3*, +YCpLac22 (CEN/TRP1)	This study
yA1428	*puf1Δ*, *puf2Δ*+*pPUF1*	MATα, *leu2*-*3*,*112*, *lys2*, *trp1*-*1*, *ura3*-*52*, *cup1*::*LEU2/PM*, *puf1*::*Neo* ^r^, *puf2*::*URA3* + pPUF1 [PUF1promoter-PUF1 ORF-HA – PUF1 3UTR in pYCpLac22]	This study
yA1436	*zeo1Δ*	MATa, *his4*-*539*, *leu2*-*3*,*112*, *trp1*-*1*, *ura3*-*52*, *cup1*::*LEU2/PM* (*PGK1pG/MFA2pG) zeo1::TRP1*	This study
yA1437	*zeo1Δ*	MATα, *leu2*-*3*,*112*, *lys2*-201, *trp1*-*1*, *ura3*-*52*, *cup1*::*LEU2/PM zeo1::TRP1*	This study
yA1438	*puf1Δ*, *puf2Δ*, *zeo1Δ*	MATα, *leu2*-*3*,*112*, *lys2*, *trp1*-*1*, *ura3*-*52*, *cup1*::*LEU2/PM*, *puf1*::*Neo* ^r^, *puf2*::*URA3 zeo1::TRP1*	This study

For growth in 96-wells plates, 75–100 µl of cells in the logarithmic phase were mixed with an equal volume of stress medium prepared in YPD or SCD in twice the final concentration. Plates were subjected to shaking at 900 rpm at 30 °C, and optical density was measured in 600 nm at 15 minutes intervals utilizing the Freedom EVO® (TECAN) system. In several cases (Fig. 2d), growth in microplates was measured using the Epoch microplate spectrophotometer (BioTek) at the indicated timepoints. Growth rate was calculated for each well based on measurements taken during the logarithmic phase. The logarithmic phase was defined as OD_600_ > 0.055 and lower than half of the maximal OD measured for that well. Additionally, it was considered that the logarithmic phase ended if an OD decrease between two consecutive measurements occurred more than five times. Fit of the data to the curve was considered valid if R^2^ > 0.9 and RMSE <0.2.

### Northern analysis and RNA sequencing

RNA was isolated by hot acidic phenol extraction protocol^44^. Northern analysis was performed as previously described^45^. For RNA sequencing, RNA libraries were generated using Illumina TruSeq RNA Library Preparation Kit v2 and sequenced on Illumina HiSeq 2500 platform. The number of reads was 15–30 million per sample. The reads were mapped to the S288c Saccharomyces cerevisiae version R64-2-1 genome using Tophat version 2.0.13^46^, with up to three mismatches allowed per read. The percentage of uniquely mapped reads was 87–90 per sample. Only uniquely mapped reads were counted to genes using HTSeq-count package version 0.6.1 with the ‘union’ mode^47^. Normalization was conducted using DESeq2 R package version 1.8.1^48^.

### Statistical analyses

Unclustered RNA-seq replicates similarity was calculated using Spearman correlation and was >0.95 for most cases. Only one replicate was lower than 0.9 (0.87). Differential expression analyses were conducted using ‘DESeq2′ R package^48^. Alternatively, genes with less than 100 reads in any sample for experiments 1–3 (Supplementary Table 2) were filtered out, and a combination of all three replicates (5,034 genes) was used as a reference dataset for GO analyses. The ratio of normalized expression in the T_45_/T_0_ and the T_end_/T_0_ was calculated in the wild-type strains, and differentially expressed genes were defined as those that deviated by more than two standard deviations from the mean ratio. In order to assess the knockouts effect, the ratio of normalized expression in the T_45_/T_0_ and the T_end_/T_0_ was calculated for the five strains. Scatter plots were made for all possible combinations, and the distance of each gene from the best-fit linear trend line was calculated. Differentially expressed genes were those with distances higher than the two standard deviations values for the population.

### RNA-seq data accession

RNA-seq data for all 49 samples was uploaded to the European Nucleotide Archive (ENA), with the Primary Accession PRJEB19283 (sample group ERG011854).

## Electronic supplementary material

Supplementary Figures

Supplementary Table S1

Supplementary Table S2

Supplementary Table S3

Supplementary Table S4

Supplementary Table S5
