# Supplementary material for: Identification and characterization of roles for Puf1 and Puf2 proteins in the yeast response to high calcium
Source: Sci Rep. 2017 Jun 8;7:3037. doi: 10.1038/s41598-017-02873-z (PMC5465220; doi:10.1038/s41598-017-02873-z)
Supplement: Supplementary file 1 — Supplementary Figures [file 41598_2017_2873_MOESM1_ESM.pdf]

Identification and characterization of roles for Puf1 and Puf2 proteins in the yeast response to high calcium

Ofir Haramati, Anastasia Brodov, Idan Yelin, Avigail Atir-Lande, Nitzan Samra and Yoav Arava*

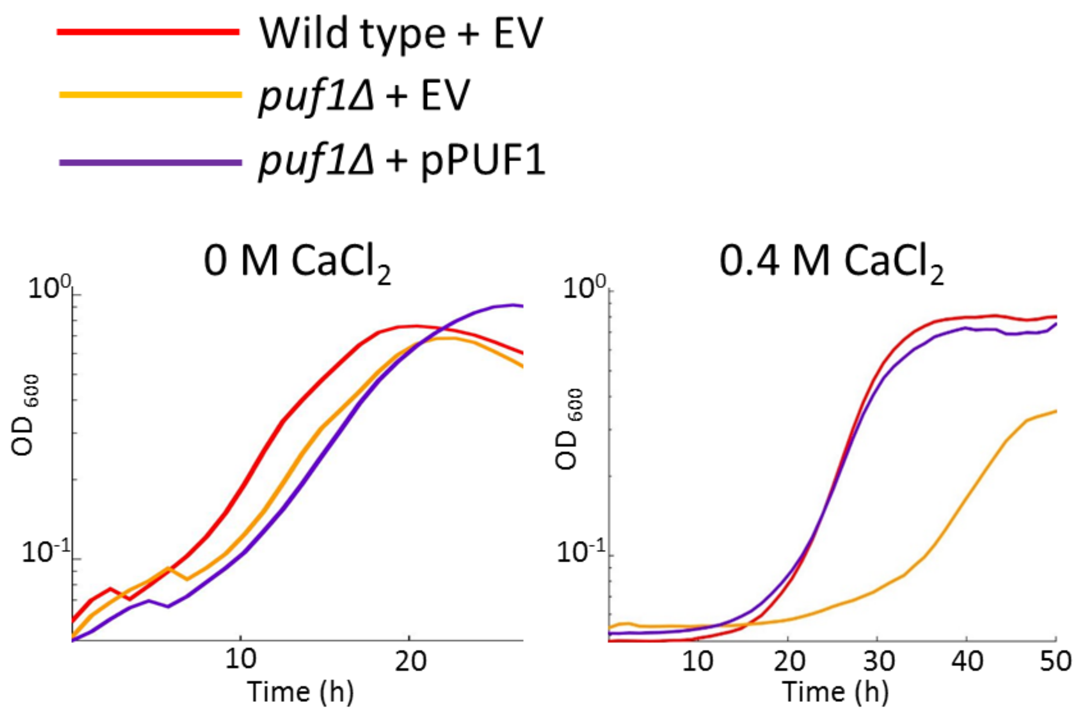

Supplementary Figure S1. Puf1 rescues the slow growth phenotype of the *puf1Δ* strain. Parental strain with an empty vector (WT + EV; pRS416), *puf1Δ* + EV and *puf1Δ* with pPUF1p (pRS416 carrying Puf1 ORF with its native promoter and 3'UTR), were grown to mid-log phase, diluted to OD600=10-2 and 200 µl were aliquoted to 96-wells plates with the proper selection growth media and with the indicated $CaCl_2$ concentrations. OD600 was measured every 15 min.

A

Gene	Time point	Strain	Ratio	
			RNAseq	Northern
YDR222W	T_{45}/T_0	Wild type	1.23	0.54
		puf1Δ2Δ	0.66	0.61
	T_{end}/T_0	Wild type	2.11	0.81
		puf1Δ2Δ	4.12	1.17
	T_{end}/T_0	Wild type	0.73	0.65
		puf1Δ2Δ	4.04	5.51
FET3	T_{45}/T_0	Wild type	3.45	3.32
		puf1Δ2Δ	0.26	0.19
	T_{end}/T_0	Wild type	4.95	1.56
		puf1Δ2Δ	2.41	0.35
	T_{end}/T_0	Wild type	1.58	1.20
		puf1Δ2Δ	0.64	0.51
ZEO1	T_{45}/T_0	Wild type	0.40	0.42
		puf1Δ2Δ	0.94	0.67
	T_{end}/T_0	Wild type	0.38	0.16
		puf1Δ2Δ	1.10	0.37
	T_{end}/T_0	Wild type	1.16	1.01
		puf1Δ2Δ	3.30	4.59
INM1	T_{45}/T_0	Wild type	1.41	0.90
		puf1Δ2Δ	1.42	1.06
	T_{end}/T_0	Wild type	1.71	0.59
		puf1Δ2Δ	0.80	0.16
	T_{end}/T_0	Wild type	0.89	0.64
		puf1Δ2Δ	0.73	1.11
PMP3	T_{45}/T_0	Wild type	2.20	2.77
		puf1Δ2Δ	1.74	1.63
	T_{end}/T_0	Wild type	1.55	1.14
		puf1Δ2Δ	2.39	0.76
	T_{end}/T_0	Wild type	0.91	0.94
		puf1Δ2Δ	0.62	0.86
FTR1	T_{45}/T_0	Wild type	0.43	1.32
		puf1Δ2Δ	5.28	9.85
HMX1	T_{45}/T_0	Wild type	0.17	0.06
		puf1Δ2Δ	0.78	0.96
SIT1	T_{45}/T_0	Wild type	0.10	0.19
		puf1Δ2Δ	0.80	0.97

B

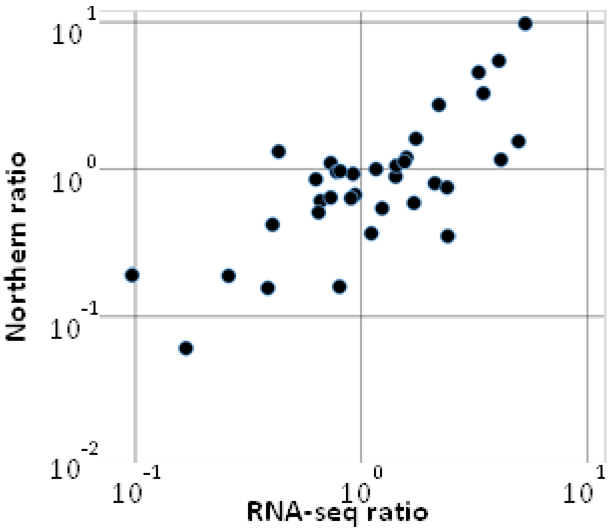

Supplementary Figure S2. Northern validation of RNA-seq data. (A) Relative expression for the indicated genes was determined by Northern analyses for the indicated strains. Signals were normalized to the signals before application of CaCl2 (T_0). The T_0-normalized signals that were obtained by the RNA-seq analysis are also indicated. Some genes have replicate data from independent biological repeats. (B) Scatter plot of Northern blot ratios against RNA-seq ratios (log scale). Compatibility of the results calculated using t-test (Correlation=0.73; two-tailed *p* value=4.3E-07)
